# Unsatisfactory Post-operative Imaging Is Predictive of Revision Surgery in Intracapsular Hip Fracture Fixation

**DOI:** 10.7759/cureus.63647

**Published:** 2024-07-02

**Authors:** Duncan E Whittaker, Luke Farrow, David Neilly, Sahil Gaba, Joaquim Goffin, Iain Stevenson

**Affiliations:** 1 Department of Trauma and Orthopaedics, NHS Grampian, Aberdeen, GBR

**Keywords:** hip fixation, failure, fixation, hip fracture, intracapsular

## Abstract

Failed fixation of intracapsular hip fractures in young patients is associated with high morbidity and cost. Accordingly, we set out to determine the association between unsatisfactory post-operative imaging (judged by two fellowship-trained trauma consultants and a senior trainee) and the risk of subsequent reoperation, including adjustment for potential confounding variables.

Ninety-four (94) patients aged <60 were included in the study from a single major trauma centre. Exhausted patients (19%) required further surgery, with the most common reason being avascular necrosis (n=10) followed by non-union (n=6). Univariate analysis found only smokers and unsatisfactory fracture reduction to be predictive of failure (p < 0.05). Other demographics and recognised scoring systems from the literature were poor predictors of failure apart from the Haiduewych classification system, utilised to assess the quality of reduction, which showed a trend towards significance (p0.053). Multivariate analysis showed smoking and unsatisfactory fracture reduction to be strong predictors of failure (p<0.05). In those with unsatisfactory fracture reduction, 50% required reoperation compared to 17.5% of those with satisfactory reduction.

This study highlights the key principles of ensuring adequate intraoperative reduction and fixation, in keeping with GIRFT principles. Avoiding secondary reoperation is crucial to prevent long-term negative outcomes for this typically high functional demand group of patients. We recommend regular, consultant peer review of post-operative imaging as a method of identifying substandard fracture fixations and those at risk of failure. This will allow opportunities for teaching, clinical improvement, and multidisciplinary team (MDT) discussions of at-risk patients.

## Introduction

Intracapsular hip fractures are the leading cause of death following accidents with 30-day mortality in the UK being 6.2% in 2022 [1]. The management and complications associated with these injuries can lead to prolonged hospital admission and have an associated healthcare cost of approximately £1 billion per year [2]. National Institute for Health and Care Excellence (NICE) guidelines recommend that patients over the age of 60 with displaced intracapsular fractures should undergo replacement with hemiarthroplasty or total hip arthroplasty (THA) [3]. In this age group, the decision to fix or replace undisplaced or valgus-impacted fractures remains one of debate. In 2011, Parker M et al. performed a Cochrane Review comparing the outcomes of internal fixation versus arthroplasty. They concluded fixation led to a shorter length of surgery and lower operative blood loss but was also associated with an increased risk of re-operation when compared to arthroplasty (40% versus 11%) [4]. This was later explored by the fracture fixation in the operative management of hip fractures (FAITH) trial in 2017. They performed a multi-centre, randomised control trial, comparing sliding hip screws to cancellous screw fixation in patients aged over 50, using reoperation at 24 months as their primary outcome. They found no statistical difference between the two groups (20% and 22%, p=0.18); however, did note that sliding hip screw fixation performed better in displaced fractures, basicervical fractures and in smokers although only the smokers were found to be statistically significant. The search inclusion criteria were limited to low-energy injuries only and for those over age 50 [5].

Most high-level literature has focused on displaced intracapsular hip fractures in patients aged over 60. It is generally accepted in the young that fixation is favoured due to the biomechanical benefits of maintaining the native femoral head. However, there is a developing consensus that total hip arthroplasty (THA) offers improved functional outcomes and lower rates of complications compared to internal fixation in the correct patient population [6]. This has been attributed to the avoidance of the serious complications of fixation such as non-union and avascular necrosis, which have been shown to be 9% and 23%, respectively [7]. This, however, should be offset by the risk of aseptic loosening, which has been shown in both cemented and uncemented THA. It is, therefore, important to reflect and understand our current practices by analysing the risk factors that lead to reoperation in this functionally high-demand group of patients.

In this study, we aim to evaluate the outcomes of all patients under 60 who underwent hip fracture fixation in the Northeast of Scotland between August 2013 and December 2019. We aim to determine any variables that led to fixation failure, including the application of recognised scoring systems and the addition of our own retrospective imaging review.

## Materials and methods

From our prospective trauma database, we identified all patients under the age of 60 with intracapsular neck of femur fractures that underwent hip fracture fixation between August 2013 and December 2019. Age <60 was chosen as the consensus among the authors was this provided a good catchment of the physiological “young elderly”. December 2019 was chosen to provide a minimum of two years follow-up. Patients were excluded if they presented more than one week following injury, if they had previous surgery to the affected hip or if the aetiology was atraumatic such as a stress or pathological fracture. Using this criteria, 94 patients were identified.

Patient’s electronic notes were reviewed (determined via prior analysis of relevant literature regarding hip fracture fixation failure) and demographic data was collected on patient age, sex, smoking and alcohol status at the time of their injury. The injury mechanism was recorded as either low energy (fall from standing) or high energy. Time to fixation was recorded as <24 hours or > 24 hours after X-ray confirmation of injury. Anaesthetic notes were reviewed for the patient's American Society of Anesthesiologists (ASA) score.

Post-operative imaging was blindly reviewed by two fellowship-trained trauma consultants and one senior trainee with a trauma subspecialty interest who acted to help with conflict resolution. Fracture reduction was given a rating of satisfactory or unsatisfactory. Thereafter, pre, intra and post-operative X-rays were reviewed and recognised scoring classifications were then applied. These included the Garden, Pauwel and Haidukewych classifications [8-10]. For implants using a sliding hip screw, tip-apex-distance (TAD) was calculated using Baumgartners methodology [11].

The minimum follow-up was two years or until fracture union. The radiological union was confirmed on X-ray in the follow-up clinic. The primary outcome measure was reoperation defined as surgery that occurred subsequent to the initial procedure to relieve pain, treat infection or improve function. Secondary outcomes were fracture complications, including avascular necrosis (AVN), non-union, implant failure and infection.

Data analysis

Data analysis was performed using IBM SPPS 24 for Windows (IBM Corp., Armonk, NY, US). Bivariate logistic regression was used to determine the risk of reoperation for the pre-operative variables age, sex, smoking status, alcohol status, energy mechanism, American Society of Anesthesiologists (ASA) score and time to fixation. Logistic regression was also performed on the operative variables, which included Garden, Pauwel and Haidukewych classifications, implant type and TAD. Multivariate logistic regression was then performed on significant or near-significant variables to determine which factors were independent predictors of failure. Given the limited sample size, the multivariate analysis was limited to three variables to avoid overfit.

Variables were grouped so they could be bivariate. This was done following a review of the literature and the common groupings used. Age was separated, split at <50 / >50, the Garden classification into 1-2 / 3-4, Pauwels 2 + 3 (there was no Pauwel 1 in the group that failed), Haidukewych 1-2 / 3-4 and ASA 1-2 / 3-4. TAD was split at <25 mm / >25 mm. The significance level was reported at p=0.005.

## Results

Using the initial search strategy, 99 potential patients were identified for the study. Of these, four were excluded; two due to undergoing primary arthroplasty, one as the indication was a stress fracture and one as we deemed the fracture to be extracapsular. Of the remaining 94 patients, 18 (19%) required further surgery.

The case mix variable can be seen in Table 1. Of the 94 patients, there were 57 males (61%) and 37 females (39%), with a mean age of 47 and 52 years old, respectively. Fifty-two per cent (52%; n=49) of injuries were deemed as high energy with an average age of 47. 85% (n=80) of patients were operated on within 24 hours of their fracture diagnosis. Only two types of implant were used: a sliding hip screw plus a two-hole plate or three cannulated screws. The blinded consultant rating and Haidukewych scoring system were unable to be performed on five patients due to the lack of post-operative X-rays.

**Table 1:** Case mix variables for the study cohort (n=94) n: number of cases, %: percentage of cases in the study cohort, ASA: American Society of Anesthesiologists

Case-mix variable	n (%)
Gender	
Male	57 (61)
Female	37 (39)
ASA	
I	40 (43)
II	45 (48)
III	9 (9)
IV	0 (0)
Smoking status	
Non-smoker	62 (66)
Smoker	32 (34)
Alcohol	
Safe consumption	83 (88)
Excess consumption	11 (12)
Energy mechanism	
Low	45 (48)
High	49 (52)
Side	
Right	43 (46)
Left	51 (54)
Time to surgery	
<24 hours	80 (85)
>24 hours	14 (15)
Surgical implant	
Cannulated screws	17 (18)
Sliding hip screw with 2-hole plate	77 (82)
Garden classification	
I	2 (2)
II	17 (18)
III	26 (28)
IV	49 (52)
Pauwel classification	
I	13 (14)
II	54 (57)
III	27 (29)
Haidukewych	
I	32 (36)
II	35 (39)
III	17 (20)
IV	6 (7)
Tip-apex distance	
<25 mm	69 (90)
>25 mm	8 (10)
Fracture reduction	
Satisfactory	80 (89)
Unsatisfactory	10 (11)
Further surgery	
Arthroplasty	15 (83)
Girdlestone	2 (11)
Removal metalwork	1 (6)

Eighteen (19%) patients required further surgery with the most common reason being avascular necrosis (n=10) followed by non-union (n=6). This involved 15 arthroplasty procedures, 2 Girdlestones and 1 removal metalwork. Fifty per cent (50%) of patients who required further surgery were smokers (n=9).

Univariate analysis only showed both smokers (OR 3.068, 95% CI 10.070; 8.799, p=0.037) and unsatisfactory fracture reduction (OR 5.154, 95% CI 1.304;20.375, p=0.019) to be predictive of failure. In multivariate analysis, smoking (OR 0.264, 95% CI 0.036; 0.668, p=0.023) and unsatisfactory fracture reduction (OR 0.154, 95% CI 0.036; 0.668, p=0.012) were found to be strong predictors of failure (Table 2).

**Table 2:** Effect of case-mix variables on fracture fixation failure BR: bivariate logistic regression analysis, MR: multivariate logistic regression analysis, ASA: American Society of Anesthesiologists, OR: odds ratio, 95% CI: confidence interval set at 95%, p value significance <0.05

	BR OR (95% CI)	p value	MR OR (95% CI)	p value
Fracture reduction	5.154 (1.304; 20.375)	0.019	0.154 (0.036; 0.668)	0.012
Smoking status	3.068 (1.070; 8.799)	0.037	0.264 (0.084; 0.832)	0.023
Age	0.688 (0.233; 2.026)	0.497	0.560 (0.172; 1.84)	0.560
Gender	0.726 (0.246; 2.140)	0.561	NA	NA
Haidukewych	2.841 (0.988; 8.170)	0.053	NA	NA
Pauwel	1.497 (0.653; 3.433)	0.341	NA	NA
Garden classification	0.190 (0.024; 1.525)	0.118	NA	NA
Side	0.527 (0.179; 1.549)	0.244	NA	NA
Tip-apex distance	3.540 (0.729; 17.195)	0.117	NA	NA
High energy	2.222 (0.756; 6.534)	0.147	NA	NA
ASA	1.062 (0.206; 5.491)	0.942	NA	NA
Alcohol excess	2.816 (0.726; 10.931)	0.135	NA	NA
Implant choice	0.488 (0.147; 1.621)	0.241	NA	NA
Time to surgery	0.285 (0.035; 2.336)	0.24	NA	NA
Diabetes mellitus	2.250 (0.379; 13.365)	0.372	NA	NA

**Figure 1:**
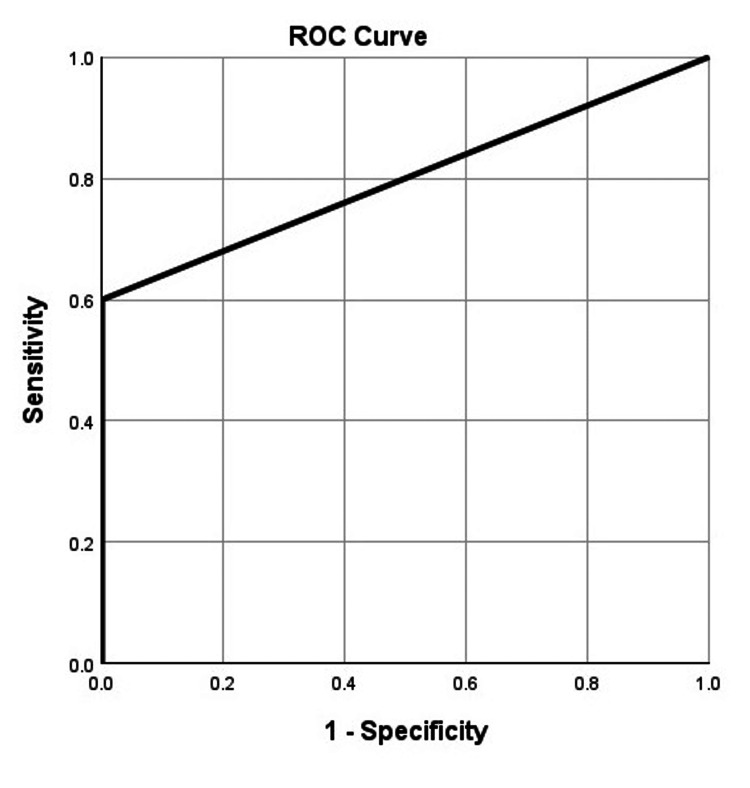
Receiver operating characteristic (ROC) area under the curve (AUC) for the model, including unsatisfactory fracture reduction and positive smoking status

In those deemed to have unsatisfactory fracture reduction, 50% (n=5) require reoperation compared to 17.5% (n=14) that were deemed satisfactory. The receiver operating characteristic (ROC) area under the curve (AUC) for a model including unsatisfactory fracture reduction and smoking status was 0.800 indicating excellent predictability (Figure 1).

## Discussion

We have demonstrated that smokers and unsatisfactory fracture reduction are independent predictors of requiring further surgical intervention in intracapsular hip fracture fixation. Smoking is a well-recognised risk factor in fracture fixation failure and our study corroborates the findings of larger studies such as the FAITH trial [5]. Fracture reduction, although not objective, highlights the importance of achieving the best possible reduction and fixation in theatre, anything less will significantly increase the likeliness of further surgery. This is a cornerstone of orthopaedic surgery but is particularly relevant to intracapsular hip fracture fixations due to the tenuous neck of femur blood supply and the propensity to cause avascular necrosis or non-union once damaged [12]. This was particularly evident in the smoking population where reoperation was three times more likely if the surgical reduction was deemed unsatisfactory.

Although subjective, the post-operative X-ray review principles are founded upon the learned experiences a surgeon develops over time. In learning theory, this is known as experiential learning. Often, upon review of intra-operative X-rays, there is no single variable that predicts failure, but a multitude of small variables that combined, predict failure. This was highlighted by the inability of recognised, post-reduction scoring systems, such as Haidukewych, to evidence statistical significance in our analysis [10]. Fracture reduction quality can be influenced by surgical experience, inadequate skill set and implant positioning. These factors are modifiable and are necessary to avoid poor fracture reductions. An experienced surgeon draws on their own past experiences and can recognise when a fixation is sub-standard and will likely fail. As shown in this study, the classic objective scoring systems used in the literature were shown to be insignificant predictors of failure [8-11]. Rajasekaran et al. highlight this message in their study of over 6000 fracture fixations. The authors organised a tri-weekly, consult-led, peer review of all fractures fixed in their tertiary trauma centre. Fracture fixations were classified into three categories; A (good fixation); B (acceptable fixation needing monitoring); and C (poor/unacceptable fixation needing immediate revision). No objective measures were applied and category conclusions were based on clinical experience and post-operative imaging review. Instigation of this peer review process led to a stepwise improvement in fracture fixations with an increase in good fixations going from 87.7% (n=5770) to 94.6% (n=6605) and unacceptable fixations dropping from 2.23% (n=243) to 0.2% (n=15) in a three-year period. The study did not provide outcome data on post-operative complications, infection or delayed revisions, but it does demonstrate a clear stepwise improvement upon the instigation of a robust consultant-led, peer review process. From their study, they were able to identify higher-risk fractures pre-operatively and allocate an appropriately experienced surgeon to perform the operation with the added motivation of peer review post-operatively [13].

Our results demonstrate the utility of senior peer review of post-operative imaging as a useful tool in identifying patients at high risk of failure and therefore candidates for early revision surgery or close monitoring in outpatient clinics. Good fixation requires an audit and focused education by training surgeons on a case-by-case basis. These findings align with the principles of the Get It Right First Time (GIRFT) initiative first published in 2015 and recently updated in 2020 [14]. These reports have driven a message of continuous and regular clinical governance leading to reduced costs, increased efficiency, and reduced litigation. The report also emphasises the importance of teaching modules in healthcare systems to achieve goals of excellence. Peer review sessions offer opportunities for collective learning, skill enhancement and multidisciplinary team (MDT) discussions, potentially reducing the incidence of fixation failure.

A theme not explored in this study was the potential alternative to hip fracture fixation in this population. We have demonstrated a 19% (n=18) failure rate, which although high, remains lower than seen in the current literature of 28-53.5% [15-19]. Swart et al. reported primary arthroplasty for displaced neck of femur fractures, in patients aged 45-65, to be more cost-effective than fracture fixation. The economic benefit was shown to increase with increasing comorbidity [20]. Historically accepted practice was to offer fixation for all intracapsular fractures under age 60 has now changed, with increasing recommendations to consider patient comorbidities and offer arthroplasty if associated risk factors for poor bone quality and failure are present [18].

A limiting factor of this study was that only patients who went on for further surgical intervention were labelled as failures. It is possible that patients with low functional demand or who died did not re-present and may have been covert failures. Furthermore, the subjective nature of assessing fracture reduction quality, despite its demonstrated predictive value, may limit reproducibility across different clinical settings. Lastly, due to the retrospective nature of the study, patients who were deemed at high risk of failure and multi-comorbid may have been excluded and managed with arthroplasty, as it is becoming more standard practice within our centre.

## Conclusions

This study demonstrates that unsatisfactory post-operative fracture reduction is a robust predictor of revision surgery in intracapsular hip fracture fixation, particularly in high-risk groups such as smokers. Avoiding secondary reoperation is crucial to avoiding long-term negative outcomes for this typically high functional demand group of patients. We recommend the instigation of regular consultant peer review of post-operative imaging as a method of identifying substandard fracture fixations and those at risk of failure. This will allow opportunities for teaching, clinical improvement and MDT discussions of at-risk patients.
